# Network Pharmacology-Based Strategy to Investigate the Pharmacologic Mechanisms of Coptidis Rhizoma for the Treatment of Alzheimer's Disease

**DOI:** 10.3389/fnagi.2022.890046

**Published:** 2022-06-21

**Authors:** Xian-wen Ye, Hai-li Wang, Shui-qing Cheng, Liang-jing Xia, Xin-fang Xu, Xiang-ri Li

**Affiliations:** ^1^Centre of TCM Processing Research, Beijing University of Chinese Medicine, Beijing, China; ^2^Beijing Key Laboratory for Quality Evaluation of Chinese Materia Medica, Beijing University of Chinese Medicine, Beijing, China

**Keywords:** Alzheimer's disease, coptidis rhizoma, network pharmacology, AD pathology, molecular docking

## Abstract

**Background:**

Alzheimer's disease (AD) is becoming a more prevalent public health issue in today's culture. The experimental study of Coptidis Rhizoma (CR) and its chemical components in AD treatment has been widely reported, but the principle of multi-level and multi-mechanism treatment of AD urgently needs to be clarified.

**Objective:**

This study focuses on network pharmacology to clarify the mechanism of CR's multi-target impact on Alzheimer's disease.

**Methods:**

The Phytochemical-compounds of CR have been accessed from the Traditional Chinese Medicine Database and Analysis Platform (TCMSP) and Symmap database or HPLC determination. The values of Oral Bioavailability (OB) ≥ 30% and Drug Like (DL) ≥ 0.18 or blood ingredient were used to screen the active components of CR; the interactive network of targets and compounds were constructed by STRING and Cytoscape platform, and the network was analyzed by Molecular Complex Detection (MCODE); Gene Ontology (GO) function, Kyoto Encyclopedia of Genes and Genomes Pathway (KEGG) and metabolic pathway enrichment of targets were carried out with Metascape, the Database for Annotation, Visualization and Integrated Discovery (DAVID) and MetaboAnalyst platform; Based on CytoHubba, the potential efficient targets were screened by Maximal Clique Centrality (MCC) and Degree, the correlation between potential efficient targets and amyloid β-protein (Aβ), Tau pathology was analyzed by Alzdata database, and the genes related to aging were analyzed by Aging Altas database, and finally, the core targets were obtained; the binding ability between ingredients and core targets evaluated by molecular docking, and the clinical significance of core targets was assessed with Gene Expression Omnibus (GEO) database.

**Results:**

19 active components correspond to 267 therapeutic targets for AD, of which 69 is potentially effective; in module analysis, RELA, TRAF2, STAT3, and so on are the critical targets of each module; among the six core targets, RELA, MAPK8, STAT3, and TGFB1 have clinical therapeutic significance; GO function, including 3050 biological processes (BP), 257 molecular functions (MF), 184 cellular components (CC), whose functions are mainly related to antioxidation, regulation of apoptosis and cell composition; the HIF-1 signaling pathway, glutathione metabolism is the most significant result of 134 KEGG signal pathways and four metabolic pathways, respectively; most of the active components have an excellent affinity in docking with critical targets.

**Conclusion:**

The pharmacological target prediction of CR based on molecular network pharmacology paves the way for a multi-level networking strategy. The study of CR in AD treatment shows a bright prospect for curing neurodegenerative diseases.

## Introduction

Alzheimer's disease (AD) is usually characterized by cognitive impairment, whose two typical pathological features are extracellular amyloid plaque and intracellular neurofibril entanglement. The number of AD patients is increasing with the advent of the aging era, and it has become one of the significant public health problems in the world (Meng F. C. et al., 2018; Beckman et al., 2021). Neuroinflammation, synaptic degeneration, oxidative stress, and loss of hippocampal neurons are important factors leading to AD (Kumar et al., 2015; Cai et al., 2016; Teruya et al., 2021). It is estimated that the number of patients may increase double in the coming decades due to the lack of effective prevention and proper treatment (Hebert et al., 2013; Scheltens et al., 2021). If the disease of AD is not controlled, it will directly impact human health and the social economy, and long-term care will also put a massive burden on the families of patients (Association, 2021). Donepezil, rivastigmine, galanthamine, and other drugs are used to interfere with the course of AD (Cai et al., 2016). They may improve poor memory, maintain basic communication skills, and address some uncontrollable behaviors in AD patients. However, these drugs have drawbacks, such as limited therapeutic efficacy and side effects (Mancuso et al., 2011). Today, medications have not been discovered to prevent cognitive impairment and improve memory, judgment, and communication skills (Zemek et al., 2014; Mehta et al., 2017). New therapeutic strategies are urgently needed (Rodriguez et al., 2021).

The history of Traditional Chinese Medicine (TCM) in treating diseases is so long as ancient Chinese history, and it has improved the quality of life of the people (Ye et al., 2020). In the present world, human beings worldwide are suffering from COVID-19 (Ye et al., 2021). TCM has made an unparalleled contribution to human beings by resisting this disaster. The use of TCM has played an essential role in reversing the situation of epidemic prevention in China and even the world (Huang et al., 2021). This seems to give us a rare opportunity, and it also reminds us that TCM may play a particular role in the prevention and treatment of AD. Many results demonstrate that TCM, which regulates autophagy, is a potential therapeutic candidate for neurodegenerative disease treatment (Wang Z. Y. et al., 2021), or attenuating Aβ and tau pathology in experimental AD models (Iyaswamy et al., 2020b). It is not surprising that there have been many reports on preventing and treating AD with TCM (Kim et al., 2017; Xu et al., 2021). For example, Yuan-Hu Zhi Tong Prescription mitigates tau pathology. It alleviates memory deficiency in the preclinical models of AD (Iyaswamy et al., 2020a), and a modified formulation of Huanglian-Jie-Du-Tang reduces memory impairments and Aβ plaques in a triple transgenic mouse model of AD (Durairajan et al., 2017).

Coptidis Rhizoma (CR), named Huang Lian, is the dried rhizome of *Coptis chinensis* Franch., C. *deltoidea* C. Y. Cheng et Hsiao or *C. teeta* Wall (Ranunculaceae). It has a significant therapeutic effect on bacillary dysentery, typhoid, tuberculosis, and other diseases (Meng F. C. et al., 2018). Berberine, palmatine, coptisine, epiberberine, jatrorrhizine, and columbine, are the main protoberberine-type alkaloids of CR (Meng F. C. et al., 2018). In recent years, CR has been made new progress in preventing and treating AD, especially its principal feature, berberine, which has become a “star molecule” (Wang et al., 2020; Liang et al., 2021). The role of berberine in the prevention and treatment of AD in antioxidation (Ahmed et al., 2015), anti-inflammation (Cai et al., 2016), and anti-endoplasmic reticulum stress have been reported in many studies (Wu et al., 2021). As we all know, TCM is a complex component system, and the material basis of CR represented by berberine alone missed accord with the holistic concept of TCM. Therefore, we propose using the network pharmacology method to explain CR's overall mechanism in treating AD from the multi-level “component-target-pathway.”

**Figure 1:**
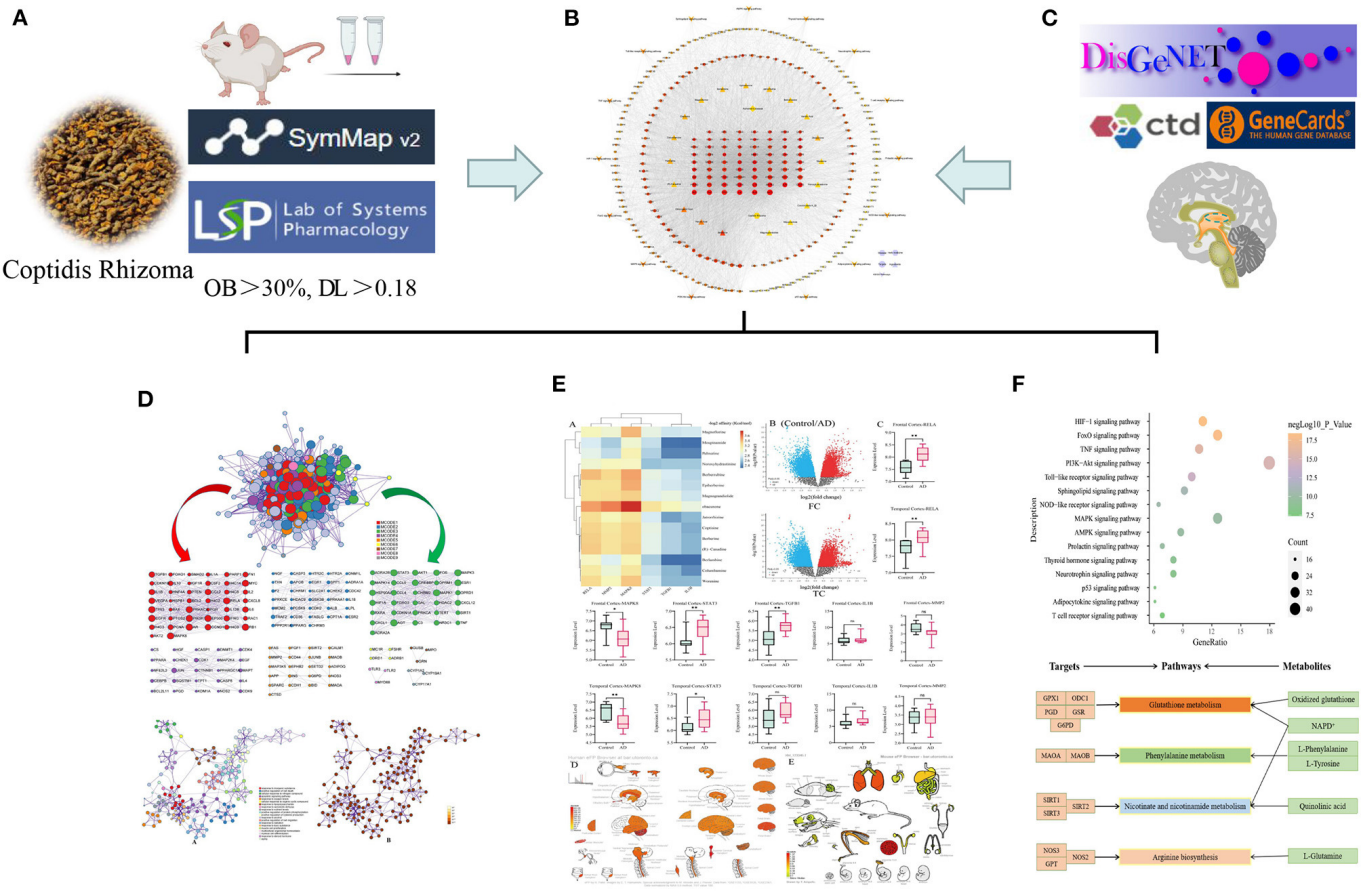
A comprehensive strategy diagram for CR in AD treatment. **(A)** Chemical composition collections for CR. **(B)** Compositions-targets network diagram. **(C)** Collection of genes related to AD. **(D)** Gene ontology enrichment (GO). **(E)** Molecular docking and analysis of core gene expression. **(F)** Pathway enrichment analysis.

In this study, the blood components of CR were selected as the research object, combined with the data of blood metabolomics and brain transcriptomes. From the view of the component-gene-metabolism level, the mechanism was expounded by utilizing network pharmacology to analyze CR in AD treatment, as shown in Figure 1. This study reveals the various means of TCMs in treating diseases from a new perspective.

## Materials and Methods

### Chemical Composition Collections

TCMSP Version 2.3 (https://tcmsp-e.com/) is a unique platform for the systematic pharmacology of TCMs, capturing the relationship between drugs, targets, and diseases (Ru et al., 2014). In this work, compounds with OB ≥ 30% are selected as the candidate molecules for further analysis (Xu et al., 2012). A molecule that gives DL ≥ 0.18 is considered a “drug-like” compound and selected as the candidate molecule for the following processes. All the compounds' properties of OB and DL are presented in the TCMSP (Tao et al., 2013). The SymMap 2.0 version (symmap.org) provides a great deal of information about herbs, ingredients, and targets related to clinical symptoms and diseases, which can be used in drug screening (Wu et al., 2018). Blood ingredients bona fide constituents are absorbed into the blood and detected. This work indicated that all the blood ingredients as the candidate molecules are presented in the SymMap. The candidate small molecules of TCMSP and SymMap are merged to construct the chemical component library of CR.

### Drug Target Collection

The Comparative Toxicogenomics Database (CTD, ctdbase.org) provides artificially selected information about the chemical, disease, and genetic relationships (Davis et al., 2021). The ingredients' targets were constructed from the TCMSP, SymMap, and CTD databases in this work. To facilitate the follow-up processing, the relevant information of the targets was sorted out uniformly with Universal Protein (UniProt, uniport.org), a comprehensive resource of protein sequences and annotation data (Consortium, 2017).

### Collection of Genes Related to AD

GeneCards version 5.6 (genecards.org) (Safran et al., 2010) and DisGeNET version 7.0 (disgenet.org) (Piñero et al., 2017) are comprehensive, user-friendly, providing information about all annotated and predicted human disease-related genes. The keyword of “Alzheimer's Disease” was searched to collect AD-related targets in GeneCards, DisGeNET, and CTD databases.

### Drug-Disease-Target Enrichment

#### Common Target Acquisition

FunRich version 3.1.3 is a compact and standalone bioinformatics analysis software, which can be used for functional enrichment and interaction network analysis of genes. With the Venn plug-in of FunRich (Pathan et al., 2015), drug-disease common targets were extracted.

#### Gene Ontology Enrichment

Enrichment refers to classifying genes according to prior knowledge, that is, genome annotation information. After gene classification, it can help recognize whether the genes found have something in common, such as function, composition, etc. A subset of enriched terms was rendered as a network plot; edges connect a similarity > 0.3 to capture the relationships between the modules further.

#### Protein-Protein Interaction Enrichment Analysis

Protein-protein interaction (PPI) network construction for common targets with STRING version 11.5 (string-db.org) (Von Mering et al., 2005). Cluster analysis is a classification method to characterize the similar attributes between targets, which defines the reliability of PPI network classification (Ye et al., 2020). Here, we performed cluster analysis of the formed PPI network by Molecular Complex Detection (MCODE) topology analysis with Metascape to find the key subnetworks and genes according to the relationship between edges and nodes in a hub network, which is convenient for downstream analysis.

#### Pathway Enrichment Analysis

##### Kyoto Encyclopedia of Genes and Genomes Pathway Enrichment

The standard targets were inputted into the DAVID online platform, and the “Homo sapiens” species were selected for KEGG pathway enrichment. Select the path in the first 15 of the *p-*value and use the ImageGP online drawing platform (http://www.ehbio.com/ImageGP/) to draw Enrichment Plot.

##### Joint-Pathway Analysis

The joint-pathway analysis module of MetobAnalyst 5.0 (https://www.metaboanalyst.ca/) combines non-targeted metabonomics with transcriptome for functional analysis at the metabolite-gene level (Pang et al., 2021). In this paper, 33 metabolites of whole blood markers in patients with dementia were used as the follow-up gene-metabolic regulatory network (Teruya et al., 2021).

### Analysis of Critical Components and Targets

#### Analysis of Potentially Important Components and Targets Based on CytoHubba

The drug-component-target-pathway-disease information obtained above was introduced into Cytoscape 3.9.0 for key component and target analysis. Using the characteristics of the CytoHubba plug-in, the network parameters are analyzed, including Maximal Clique Centrality (MCC), Degree, and so on. Take the targets that meet the requirements of MCC and Degree as potentially essential targets.

#### Analysis of Critical Potential Targets Based on Alzdata Database

AlzData (http://www.alzdata.org/) database makes an entire collection of current high-throughput omics data for AD (Xu et al., 2018). The selected targets of MCC and Degree were imported into AlzData, the function of convergent functional genomics (CFG) Rank was established, and the correlation between crucial targets and Aβ or Tau proteins was analyzed. The Aging atlas is a bioinformatics tool for studying the genetic correlation between aging and longevity (Aging Atlas, 2021). Much literature has revealed that AD is an age-related disease, and aging-related genes in this study are obtained from Aging Altas (Livingston et al., 2020). Genes related to senescence and significantly related to Aβ/Tau protein are used as critical targets.

### Molecular Docking Evaluation

The information about the core target was obtained from Uniprot. The docking site of the protein was received at the original ligand site by Pymol 2.4.0 (Schrodinger, 2015). The docking pocket site of the ligand-free protein was predicted by POCASA 1.1 (Yu et al., 2010) (http://g6altair.sci.hokudai.ac.jp/g6/service/pocasa/), and then the docking site was obtained by Pymol 2.4.0. After being treated with AutoDockTools 1.5.6 (Morris et al., 2009), all small molecules and proteins were converted to pdbqt format, then AutoDock Vina (Trott and Olson, 2010) was run for molecular docking. ImageGP drew the binding energy heat map between molecular proteins.

#### Clinical Characterization and Tissue Enrichment of Key Targets

To characterize the clinical significance of core targets, the transcriptome data of brain tissues of patients with AD and Control were searched through the GEO database. The differences in mRNA expression of core targets before and after AD were analyzed. At the same time, the expression distribution of critical targets in the typical nervous system was investigated by the Human eFP (“electronic Fluorescent Pictograph”) Browser (Patel et al., 2016), and the expression map of related genes was drawn.

## Results

### Potentially Active Components of CR

After a round of screening in TCMSP and Symmap databases, 13 and 12 components were obtained, respectively. Our research group identified six alkaloids from CR by HPLC (Supplementary File in Supplementary Material, Figure 2). Compared with the reported components in the literature (Hong et al., 2012; Wang J. et al., 2019), 19 potentially active ingredients were obtained by merging and deduplicating. Although the OB or DL does not meet the threshold value, it is shown as “Blood Ingredients” in Symmap, such as Chlorogenic acid, Magnoflorine, and Jatrorrhizine are within the consideration of our potential activity. Table 1 shows each component's physical and chemical information in TCMSP.

**Figure 2:**
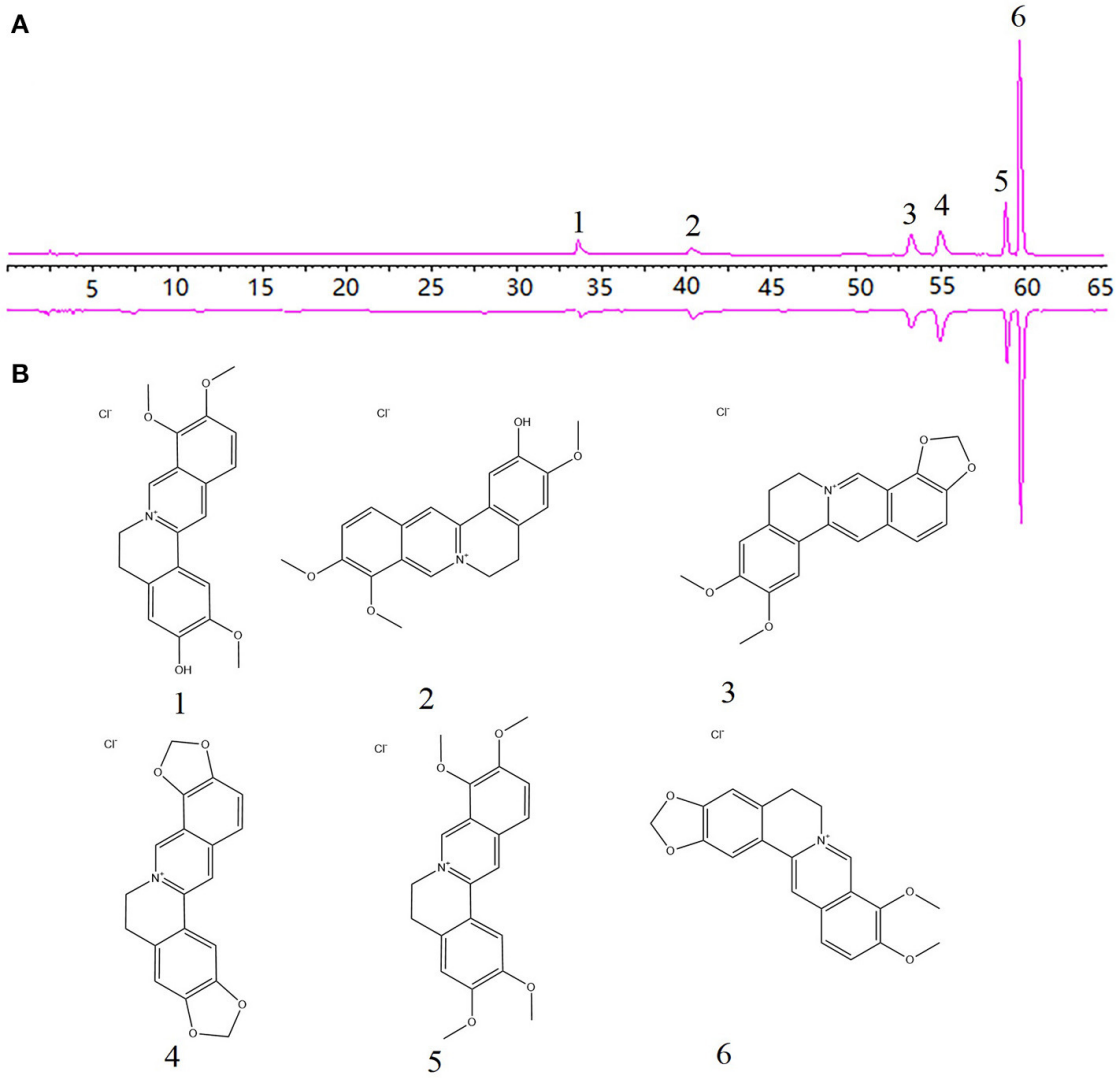
The chromatogram of 6 alkaloids from CR. **(A)** Reference solution. **(B)** Coptidis Rhizoma. (1 Jatrorrhizine hydrochloride; 2 Columbamine hydrochloride; 3 Epiberberine hydrochloride; 4 Coptisine hydrochloride; 5 Palmatine hydrochloride; 6 Berberine hydrochloride).

**Table 1:** Physicochemical information of 19 potentially active components.

**Mol ID**	**Molecule name**	**MW**	**AlogP**	**OB(%)**	**Caco-2**	**BBB**	**DL**	**FASA-**	**HL**
MOL000114	Vanillic acid	168.16	1.15	35.47	0.43	0.09	0.04	66.76	11.62
MOL000622	Magnograndiolide	266.37	1.18	63.71	0.02	−0.24	0.19	66.76	3.17
MOL000764	Magnoflorine	342.45	3.12	26.69	1.09	0.61	0.55	58.92	0.14
MOL000785	Palmatine	352.44	3.65	64.60	1.33	0.37	0.65	40.80	2.25
MOL000789	Jatrorrhizine	338.41	3.40	19.65	1.28	0.36	0.59	51.80	0.00
MOL001454	Berberine	336.39	3.45	36.86	1.24	0.57	0.78	40.80	6.57
MOL001457	Columbamine	338.41	3.40	26.94	1.01	0.11	0.59	51.80	0.00
MOL001458	Coptisine	320.34	3.25	30.67	1.21	0.32	0.86	40.80	9.33
MOL002665	Ferulic Acid	192.23	2.00	40.43	0.96	0.56	0.06	49.69	0.38
MOL002668	Worenine	334.37	3.73	45.83	1.22	0.24	0.87	40.80	8.41
MOL002894	Berberrubine	322.36	3.20	35.74	1.07	0.17	0.73	51.80	6.46
MOL002897	Epiberberine	336.39	3.45	43.09	1.17	0.40	0.78	40.80	6.10
MOL002900	Noroxyhydrastinine	191.2	0.88	38.89	0.77	0.40	0.10	47.56	8.25
MOL002903	(R)-Canadine	339.42	3.40	55.37	1.04	0.57	0.77	40.16	6.41
MOL002904	Berlambine	351.38	2.49	36.68	0.97	0.17	0.82	58.92	7.33
MOL002907	Corchoroside A_qt	404.55	1.34	104.95	−0.91	−1.31	0.78	104.06	6.68
MOL003871	Chlorogenic acid	354.34	−0.27	13.61	−1.33	−1.79	0.31	164.75	0.39
MOL008647	Moupinamide	313.38	2.86	86.71	0.55	−0.51	0.26	78.79	3.71
MOL013352	Obacunone	454.56	2.68	43.29	0.01	−0.43	0.77	95.34	−13.04

### The Targets of Potentially Active Components of CR in the Treatment of AD

The prediction targets for potentially active components were collected in TCMSP and Symmap. The reported dynamic targets of potentially active ingredients were compiled by literature mining based on the CTD database. All the targets were standardized and unified “Gene Symbol” in the Uniprot database. 65, 133, and 426 targets were obtained separately in the TCMSP, Symmap, and CTD databases. All the targets were combined to obtain 516 corresponding targets.

**Figure 3:**
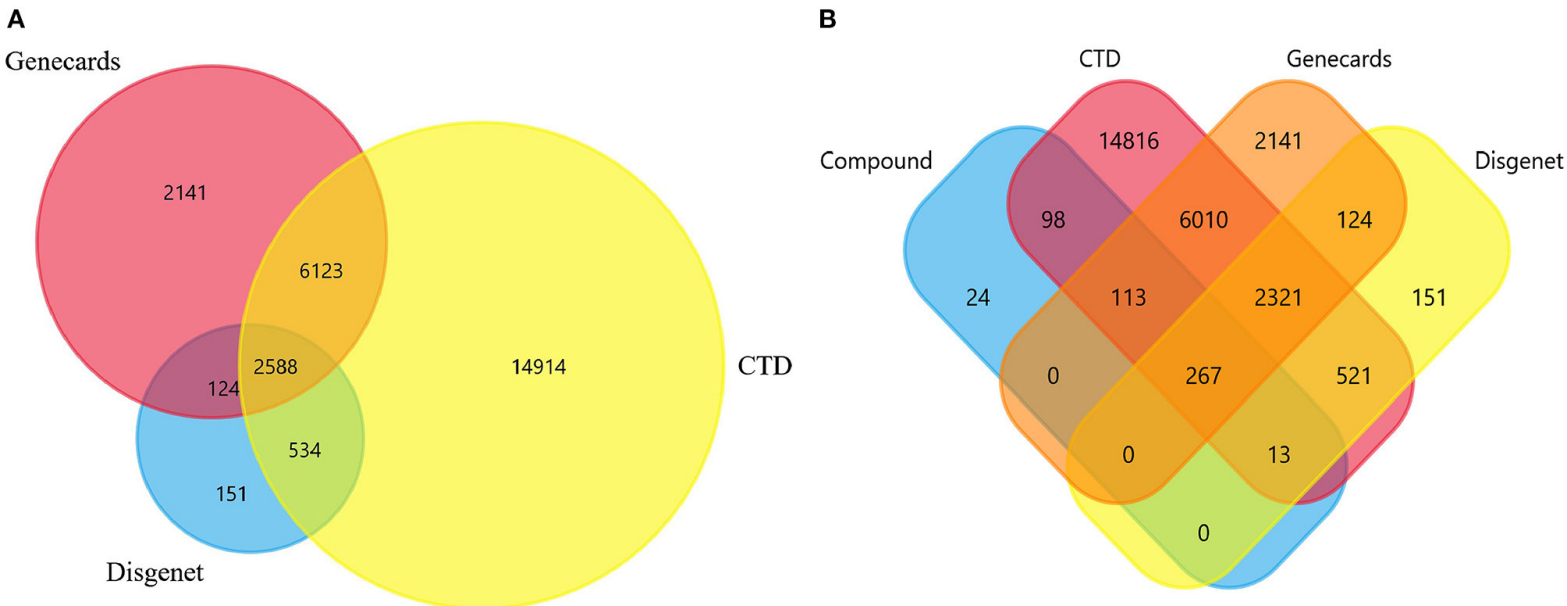
The targets of CR for the treatment of AD. **(A)** The targets of AD. **(B)** The Common targets between CR and AD.

In the CTD, DisGeNet, and GeneCards database, 24,159, 3,397, and 10,976 AD-related targets were obtained. To remove the false positive, 2,588 intersection targets were obtained by Funrich software as AD targets (Figure 3A). The acquired AD targets were intersected with the targets of potentially active components as potential therapeutic targets for CR in AD treatment (Figure 3B). Finally, we obtained 267 marks of CR for the treatment of AD.

### Enriched Ontology Clusters

**Figure 4:**
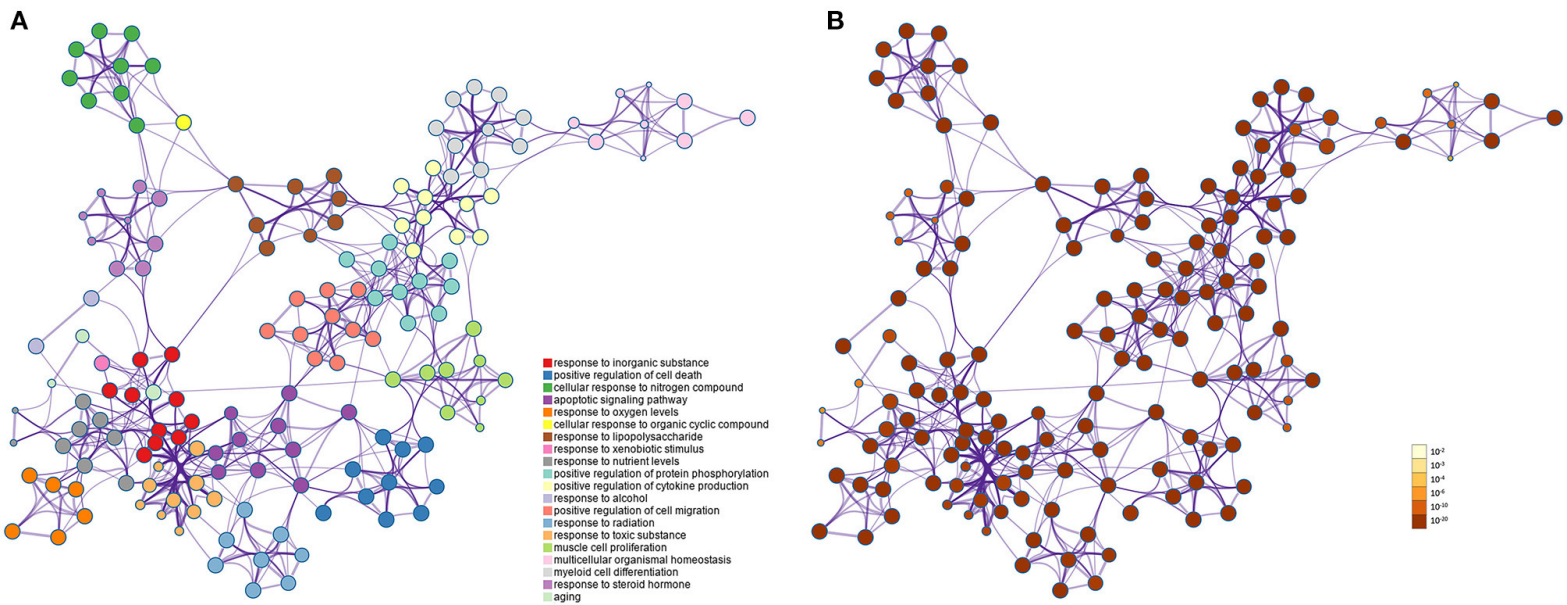
GO enrichment network map of potential therapeutic targets. **(A)** Colored by cluster-ID, the same color represents participation in similar functions. **(B)** Colored by *p*-value, the picture's colors all indicate that *p* < 0.05.

In the GO rich set, we get the results of a biological process (BP) 3,050, cellular component (CC) 184, and molecular function (MF) 257. Only the first 20 BP were shown in Figure 4. The first is the BP, which represents an orderly combination of molecular functions to achieve a broader range of biological processes, such as response to an inorganic substance, positive regulation of cell death, cellular response to nitrogen compound, apoptosis signaling pathway, response to oxygen levels, and aging. The second is the CC, which describes subcellular structures, locations, and macromolecular complexes, such as membrane raft, neuronal cell body, neuron spine, endoplasmic reticulum lumen, etc. The third is MF, which describes the functions of genes and gene products, such as antioxidant activity, superoxide dismutase activity, oxidoreductase activity, and acting on superoxide radicals as acceptors. The network is visualized using Cytoscape, where each node represents an enriched term and is colored first by its cluster ID (Figure 4A) and then by its *p*-value (Figure 4B).

### PPI Enrichment and MCODE Analysis

**Figure 5:**
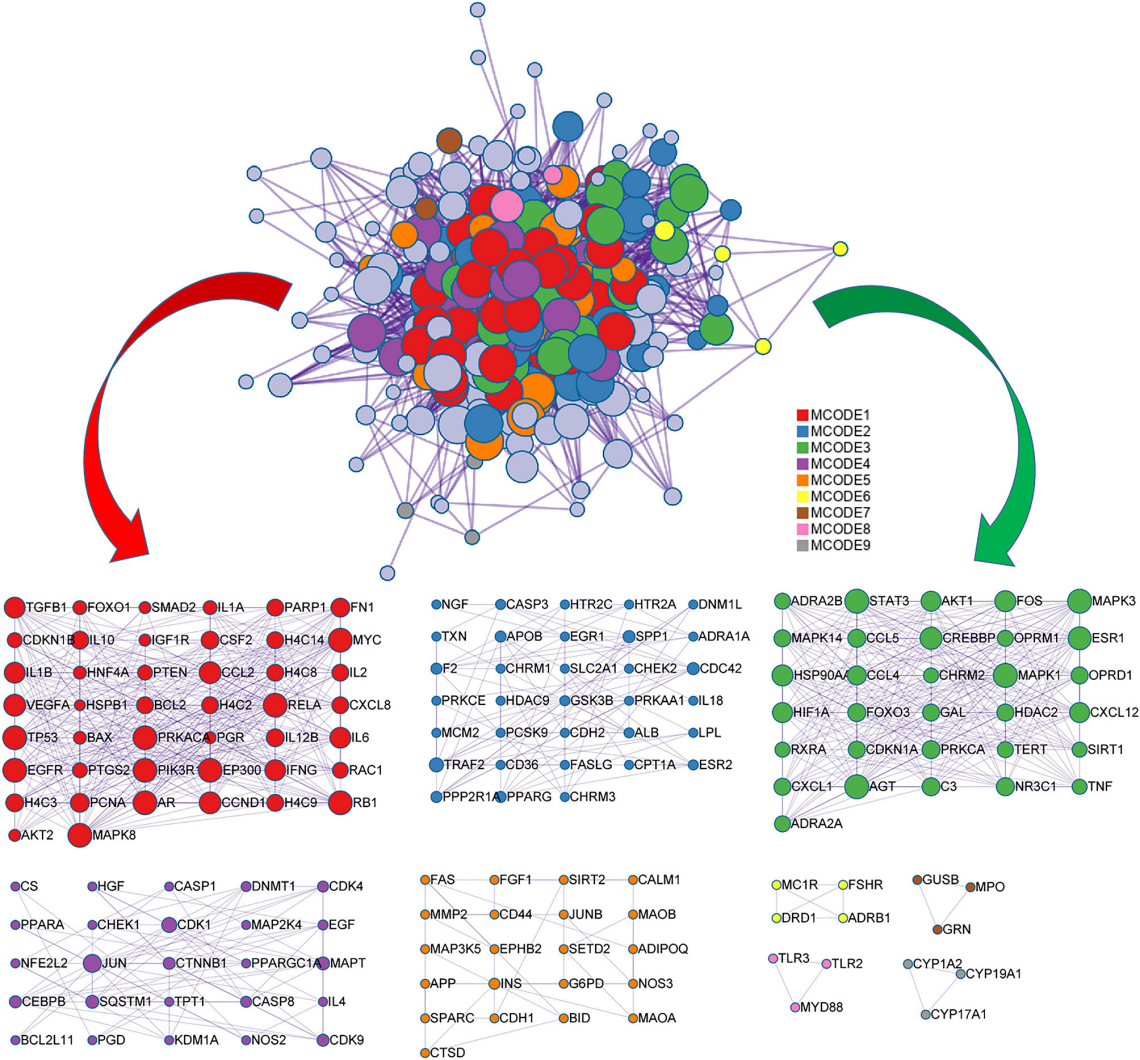
The MCODE networks identified for genes.

**Figure 6:**
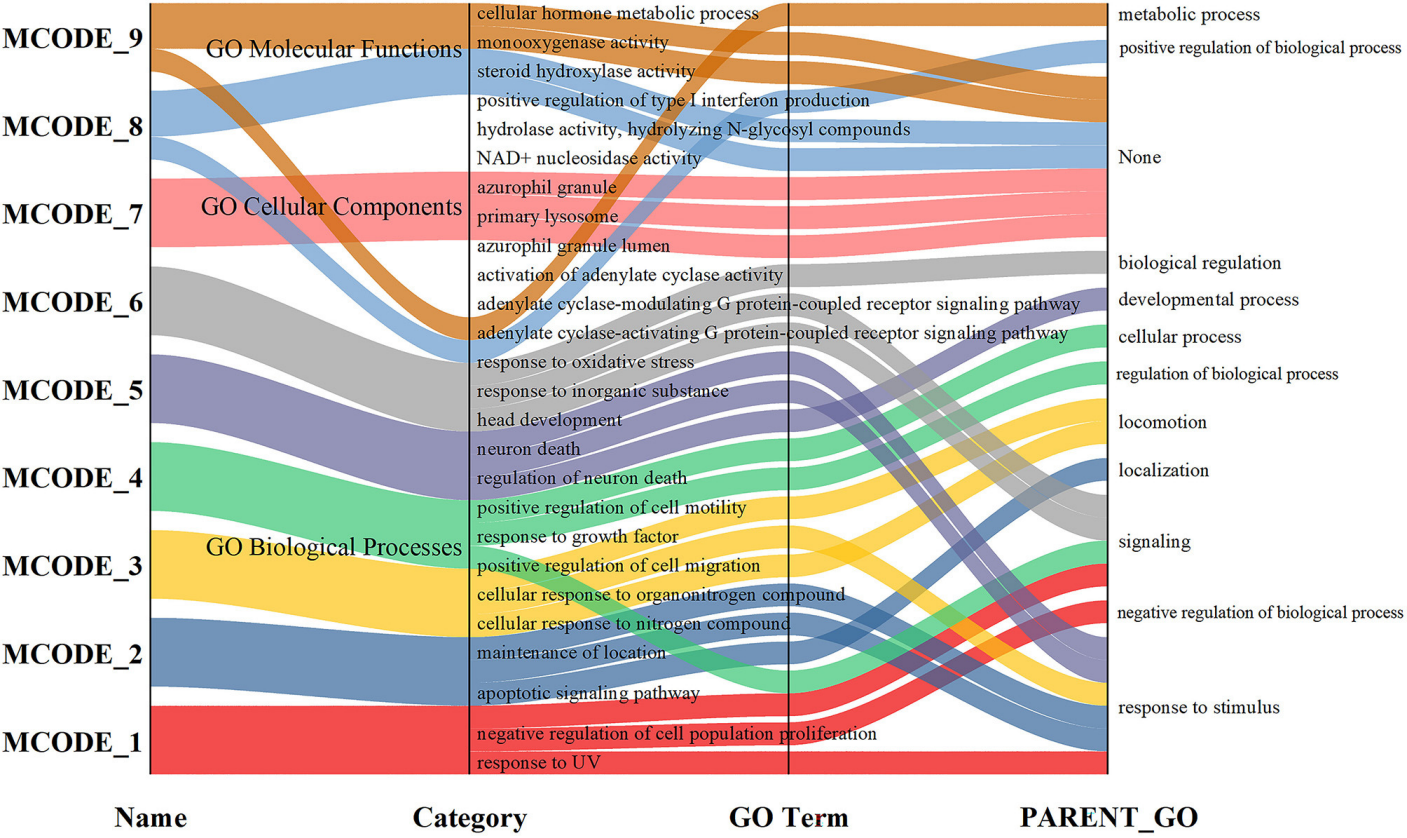
The functional description of the corresponding module.

PPI shows the interaction between targets and illustrates an interrelated information transmission network, where a PPI network diagram of node 256 and edge 6713 is shown in Figure 5. An MCODE analysis based on the PPI network diagram was carried out to classify the targets. Process enrichment analysis has been applied to each MCODE component independently. The three best-scoring terms by *p*-value were shown underneath corresponding network plots in Figure 6. In the MCODE network topology analysis, 9 analysis modules were obtained. The first module takes RELA as the center, and this module mainly participates in response to UV, negative regulation of cell population proliferation, and apoptotic signaling pathway. The second module takes TRAF2 as the center, and the module mainly participates in the maintenance of location, cellular response to nitrogen compound, and cellular response to the organonitrogen mixture. The center of the third module is STAT3. The module mainly participates in positive regulation of cell migration, response to growth factors, and positive regulation of cell motility. The center of the fourth module is JUN. The module mainly participates in the apoptotic signaling pathway, regulation of neuron death, and neuron death. Module 5 takes INS as the center, and this module mainly participates in head development, response to inorganic substances, and response to oxidative stress. In other modules, as shown in Figure 6, the enrichment results are consistent with the pathogenesis described by AD in the brain, nerve cell apoptosis, oxidative stress, and so on, indicating that the enrichment results are very reliable.

### Analysis of KEGG Enrichment and Metabolic Regulation

**Figure 7:**
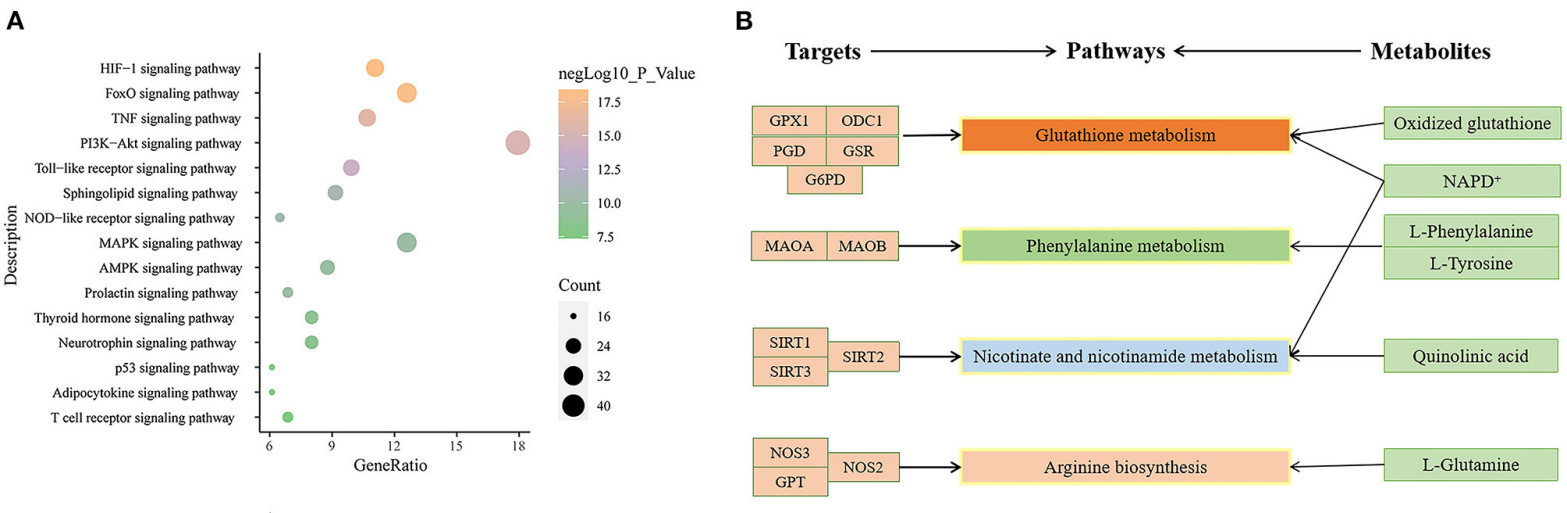
Pathway enrichment. **(A)** KEGG Pathways; **(B)** Joint pathway analysis.

In KEGG enrichment, 134 results were obtained. Many disease pathways are involved in this enrichment, such as Non-alcoholic fatty liver disease (NAFLD), Hepatitis B, Chagas disease (American trypanosomiasis), Tuberculosis, Pertussis, Influenza A, and so on, suggesting that there are similar mechanisms in different conditions. In the following Figure 7A, we showed the enrichment of the top 15 signaling pathways, which are the HIF-1 signaling pathway, the PI3K-Akt signaling pathway, the Toll-like receptor signaling pathway, the NOD-like receptor signaling pathway, the MAPK signaling pathway, the AMPK signaling pathway, and other pathways indicated that CR plays a role in the prevention and treatment of AD through multiple ways.

To explore the joint-pathway of whole blood markers in patients with dementia, 33 differential metabolites and 267 targets of blood markers in patients with dementia were analyzed by MetaboAnalyst (Figure 7B). The results showed that only Glutathione metabolism, Phenylalanine metabolism, Nicotinate, nicotinamide metabolism, and Arginine biosynthesis were simultaneously enriched with the targets from network pharmacology and the differential metabolites from metabolomics. Considering the number of metabolites and targets in the joint pathway, glutathione metabolism was chosen as the most critical metabolic pathway.

### Construction and Analysis of Component-Target-Pathway Network

#### Construction of the Component-Target-Pathway Network

**Figure 8:**
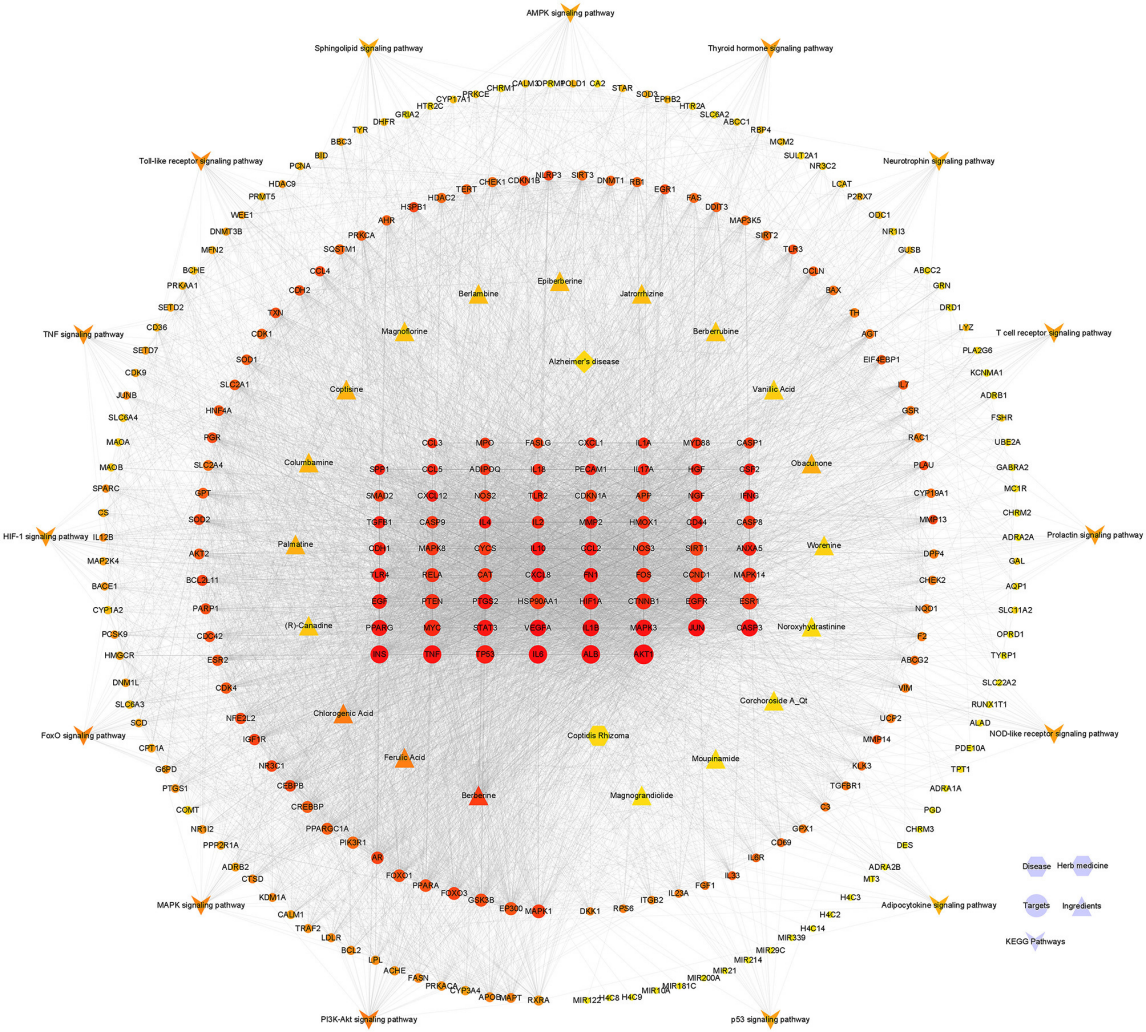
The “component-target-pathway” network diagram.

From the “component-target-pathway” network diagram (Figure 8), we can see that CR plays a role in the prevention and treatment of AD through multi “component-target-pathway.” The node's weight in the network graph is proportional to the bright degree and shape area of red. The middle circle shape is the potential core target screened by the MCC higher than “APP,” and 69 targets such as AKT1, ALB, IL6, TP53, TNF, etc. Triangle represents components, which can be judged by color. Berberine, FerulicAcid, and ChlorogenicAcid are the top three components, suggesting that these three components are the critical components of CR in preventing and treating AD.

#### Enrichment Analysis of Core Targets Based on the Whole Network

**Figure 9:**
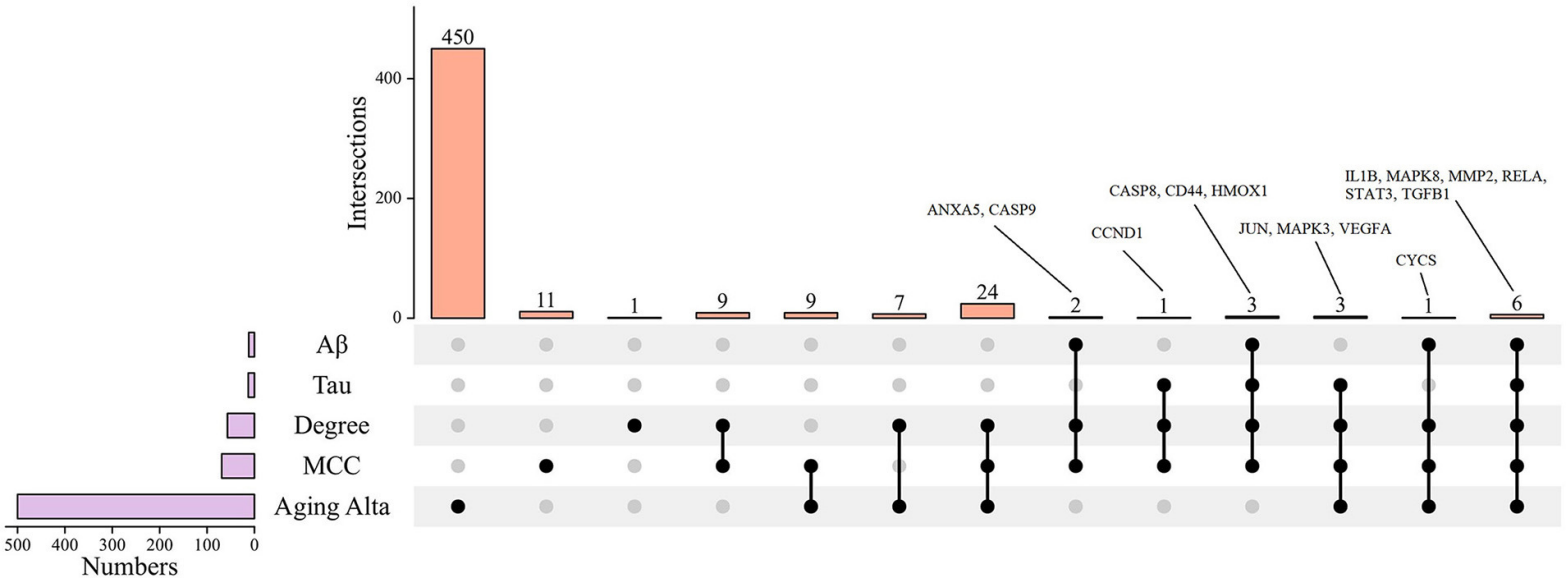
Core target enrichment map.

Taking the intersection of MCC and Degree screening targets as the following research object, 49 targets were obtained. By AlzData analysis, 10 targets were significantly positively correlated with Aβ, two were significantly negatively correlated with Aβ, Eleven targets were significantly positively correlated with Tau, and two were significantly negatively correlated with Aβ (Table 2). AD is an age-related disease. 10 age-related targets were obtained by analyzing the AgingAltas database. As can be seen from Figure 9, the six targets of IL1B, MAPK8, MMP2, RELA, STAT3, and TGFB1 are not only significantly related to Aβ and Tau but also related to age, so we speculate that these six targets are the core targets of CR for the prevention and treatment of AD (Table 3).

**Table 2:** Analysis of correlation between common targets and Aβ and Tau proteins.

**Gene**	**Pathology cor (abeta)**	**Pathology cor (tau)**
CASP1	0.488^***^	0.625^*^
CASP8	0.844^***^	0.756^**^
CCL3	0.866^***^	0.862^***^
CCL5	0.672^***^	0.794^***^
CD44	0.719^***^	0.793^***^
HMOX1	0.479^**^	0.716^**^
IL1A	0.800^***^	0.788^***^
IL1B	0.605^***^	0.844^***^
MAPK8	−0.566^***^	−0.714^**^
MMP2	0.689^***^	0.536^*^
MYD88	0.796^***^	0.769^***^
RELA	0.750^***^	0.579^*^
STAT3	0.873^***^	0.572^*^
TGFB1	0.871^***^	0.681^**^
TLR2	0.904^***^	0.755^**^
CASP9	0.493^***^	/
ANXA5	0.730^***^	/
CXCL12	0.432^**^	/
CYCS	−0.491^***^	/
VEGFA	/	−0.758^**^
CCND1	/	0.564^*^
CXCL1	/	0.663^**^
JUN	/	0.601^*^
MAPK3	/	0.686^**^
NOS2	0.327^*^	/
SMAD2	−0.369^*^	/
SPP1	0.442^**^	/

* *p < 0.05*,

** *p < 0.01*,

*** *p < 0.001*.

**Table 3:** The information on six core targets in Aging Altas.

**Symbol**	**Description**	**Function**	**Gene_Set**	**KEGG_Name**
IL1B	Interleukin 1 beta	Aged adipose B cells (AABs) express IL-1R, and inhibition of IL-1 signaling reduces their proliferation and increases lipolysis in aging.	Senescence-associated secretory phenotype	MAPK signaling pathway
MAPK8	Mitogen-activated protein kinase 8	MAPK8, also known as JNK1, encodes many transcripts and plays a critical stress response player. Overexpression of JNK in roundworms also increases lifespan.	Deregulated nutrient sensing	Insulin signaling pathway
MMP2	Matrix Metallopeptidase 2	Ubiquitous metalloproteinases are involved in various functions, such as vascular remodeling, angiogenesis, tissue repair, tumor invasion, inflammation, and atherosclerotic plaque rupture.	Senescence-associated secretory phenotype	Endocrine resistance
RELA	v-rel avian reticuloendotheliosis viral oncogene homolog A	RelA controls inducible, but not basal, transcription in NF-kappa B-regulated pathways.	Altered intercellular communication	Longevity regulating pathway
STAT3	Signal transducer and activator of transcription 3 (acute-phase response factor)	Signal transducer and transcription activator mediate cellular responses to interleukins, KITLG/SCF, LEP, and other growth factors.	Cellular senescence	Growth hormone synthesis, secretion, and action
TGFB1	Transforming growth factor, beta 1	The variability of the TGF-beta1 gene may affect longevity by playing a role in inflame-aging.	Altered intercellular communication	Cellular senescence

### Molecular Docking and Analysis of Core Gene Expression

#### Molecular Docking Analysis

**Figure 10:**
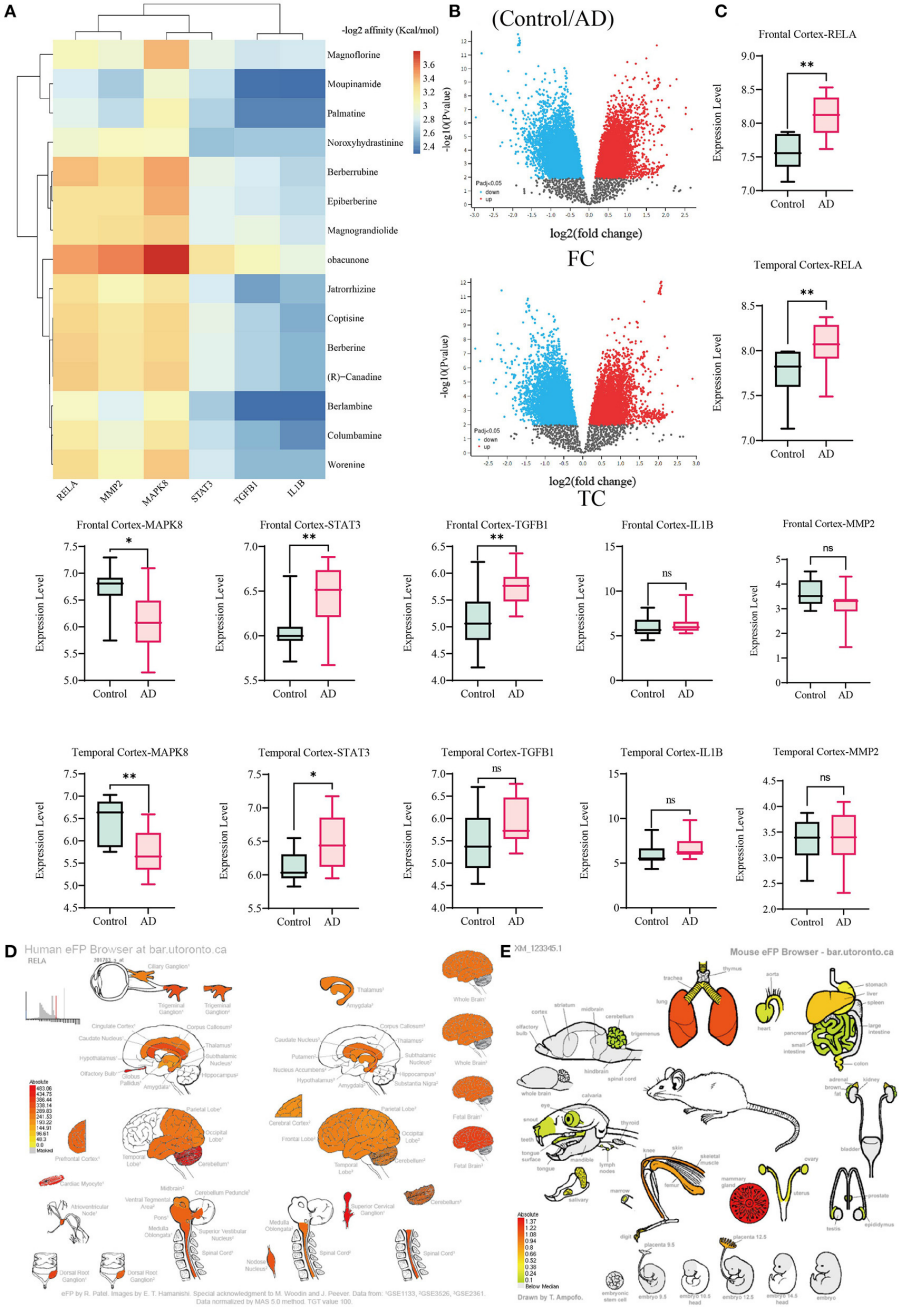
Core gene expression analysis. **(A)** Composition-Target binding energy heat map. **(B,C)** Analysis of AD Gene mRNA expression based on GEO dataset. **(D)** Analysis of the expression of RELA in the regular human nervous system. **(E)** Analysis of RELA expression in tissues and organs of normal mice. *p < 0.05, **p < 0.01.

The core target information collected by Uniprot is shown in Table 4. After removing some components that have not been successfully docked, we have drawn a composition-target binding energy heat map. In general, if the binding energy is <-1.2 Kcal/mol or −5 kJ/mol, the docking result is feasible, and there is a good affinity between the molecular targets. As shown in Figure 10A, the binding energies between the molecular targets are all <-1.2 Kcal/mol, indicating that the main components of CR have a solid crucial ability to the core targets. In terms of overall binding energy, the scores of targets MAPK8, RELA, and MMP2 are relatively high. The total binding energy of components such as Berberine, Obacunone, and Epiberberine with the target is also higher, suggesting that these components-targets may play a vital role in AD treatment.

**Table 4:** The information of six core targets in Uniprot.

**Uniprot ID**	**Genename**	**Source**	**Identifier**	**Method**	**Resolution**	**Chain**	**Positions**
P01584	IL1B	PDB	1L2H	X-ray	1.54	A	117–269
P45983	MAPK8	PDB	2XRW	X-ray	1.33	A	2–364
P08253	MMP2	PDB	3AYU	X-ray	2.00	A	110–450
Q04206	RELA	PDB	6NV2	X-ray	1.13	P	39–51
P40763	STAT3	PDB	6NJS	X-ray	2.70	A	127–688
P01137	TGFB1	PDB	1KLA	NMR	N/A	A/B	279–390

#### Core Gene Expression Analysis

The GSE122063 dataset included the brain transcriptional data of vascular dementia (VaD), AD, and Controls, and one sample was repeated twice (Mckay et al., 2019). We analyzed the expression of core genes in AD (n = 12) and Controls (n = 11) Frontal Cortex (FC) and Temporal Cortex (TC). As shown in Figures 10B,C that in AD patients, the mRNA expression of RELA and STAT3 increased significantly in FC and TC, while TGFB1 only increased significantly in FC. MAPK8 decreased significantly in FC and TC, but the difference in MMP2 and IL1B between the two sites is not statistically significant. The mRNA expression of core genes in the normal nervous system was analyzed by eFP, and it was found that the core genes were expressed to different degrees in the nervous system. As shown in Figures 10D,E, RELA has almost covered expression in the human normal nervous system. It was mainly expressed in mice's mammary glands, lungs, cerebellum, and skin.

## Discussion

In this article, we analyzed the multi-level mechanism of CR in AD treatment. Nineteen components may be the potential active components of CR, and Berberine, Chlorogenic acid, Obacunone, Epiberberine, and Ferulic Acid may be the critical components; in 267 common targets, the potential effective targets are only 69. Four marks are clinically significant among the six key targets: RELA, MAPK8, STAT3, and TGFB1. 3,050 enriched BP terms, 257 enriched MF terms, and 184 enriched CC terms. The BP terms mainly include a response to an inorganic substance, positive regulation of cell death, and cellular response to nitrogen compound; the CC terms mostly have membrane raft, neuronal cell body, and neuron spine; the MF terms mainly involve antioxidant activity, superoxide dismutase activity, oxidoreductase activity. In the MCODE network topology analysis, nine analysis modules were obtained. RELA, TRAF2, STAT3, JUN, and INS were the critical targets of each module.

Various diseases may share the exact mechanism of AD, such as NAFLD, Hepatitis B, Chagas disease, Tuberculosis, etc. Among the significant enrichment pathways, the key pathways were the HIF-1 signaling pathway, the PI3K-Akt signaling pathway, the Toll-like receptor signaling pathway, the NOD-like receptor signaling pathway, and the MAPK signaling pathway. Hypoxia-inducible factor (HIF) regulates protein expression in biological processes, such as neurogenesis, glucose metabolism, erythropoiesis, and angiogenesis (Correia and Moreira, 2010). As we age, the oxygen transport of cells and tissues will be damaged to a certain extent, thus increasing the sensitivity of neurons to injury. Therefore, it is of positive significance to protect the hypoxic adaptability of neurons in aging (Ogunshola and Antoniou, 2009).

Experimental data show that the PI3K-Akt signaling pathway may be an essential target for AD treatment. The PI3K-Akt pathway regulates various biological processes such as cell proliferation, movement, growth, survival, and metabolism and inhibits different neurotoxic mechanisms (Kumar and Bansal, 2022). In addition, the PI3K/Akt signaling pathway depends on the cellular environment. In short, PI3K/Akt is beneficial to activating neurons and neural stem cells, but the activation of microglia may be disadvantageous to the internal environment (Razani et al., 2021).

Neuroinflammation can occur in inflammatory diseases of the central nervous system and may develop into neurodegenerative diseases (Li et al., 2021). The NOD-like receptor is an essential cytoplasmic pattern recognition receptor, which plays a vital role in the host's innate immune response and immune homeostasis (Kong et al., 2017). Systemic inflammation can activate TLR4, NLRP3, inflammatory bodies, and complements in the brain, resulting in neuroinflammation, Aβ accumulation, synaptic loss, and nerve degeneration (Cheng et al., 2014). TNF and TNFR1 are involved in AD-related cerebral nerve inflammation and regulate the formation of Aβ through β-secretase (Yang et al., 2020). Therefore, the potential molecular mechanism of the inflammation-related signaling pathway can be used as a method for the prevention and treatment of AD.

There is evidence that the MAPK signal pathway may be involved in the pathogenesis of AD by regulating the activity of β- and γ-secretase, neuronal apoptosis, and phosphorylation of APP and Tau (Kim and Choi, 2010). AMPK can improve energy metabolism, stimulate autophagy and inhibit inflammation, while HIF-1α can promote angiogenesis and help cells adapt to harsh conditions (Salminen et al., 2016). The recovery/enhancement of autophagy has been considered a method for treating these neurodegenerative protein lesions (Benito-Cuesta et al., 2021).

In the joint analysis of metabolism and pathway, glutathione metabolism, phenylalanine metabolism, nicotinate and nicotinamide metabolism, and arginine biosynthesis were simultaneously enriched. Glutathione (GSH) is the most abundant non-protein mercaptan in cells and an important antioxidant. Compared with healthy older adults, the levels of GSH in the hippocampus of patients with mild cognitive impairment (MCI) and AD are significantly reduced (Liu et al., 2004). The decrease of brain antioxidant GSH is related to oxidative stress (Mandal et al., 2019). Under chronic inflammation, the rise of serum phenylalanine concentration and phenylalanine/tyrosine ratio (Phe/Tyr) may be related to neuropsychiatric symptoms (Wissmann et al., 2013). Immune activation in patients with AD is related to high serum phenylalanine concentration, and the disorder of phenylalanine metabolism in the hippocampus may be an essential mechanism of AD pathology (Liu et al., 2021). L-arginine (L-Arg) is the precursor of nitric oxide and polyamines. It has a variety of functions in human health, such as regulating atherosclerosis, redox stress, inflammation, and the regulation of synaptic plasticity and neurogenesis. It also plays a vital role in age-related degenerative diseases like AD (Asai et al., 2007; Yi et al., 2009).

It is worth noting that berberine can reduce the level of Aβ by altering the hyperphosphorylation of APP in human glioma H4 cells (Yu et al., 2011). In addition, berberine alleviates Tau hyperphosphorylation through PI3K/Akt/Gsk3β pathway (De Oliveira et al., 2016). Some literature has shown that berberine can reduce acetylcholinesterase activity in the cerebral cortex and hippocampus of rats with memory impairment induced by streptozotocin/ethanol, improve memory impairment and maintain essential memory ability (Patil et al., 2015). Improving the function of cholinergic neurons, reducing the abnormal phosphorylation of Tau, and clearing Aβ deposition are the essential mechanisms of CR in AD treatment.

It has been found that berberine can reduce the levels of NF-κB, TLR4, TNF-α, and IL-6 in the brain of adult male cognitive impairment rats induced by lipopolysaccharide and prevent learning and memory impairment (Sadraie et al., 2019). Berberine can also inhibit Aβ-induced neuroinflammation (Jia et al., 2012), block NF-κB signaling, and improve the learning and memory function of APP/PS1 mice (He et al., 2017). The anti-inflammatory of CR may play an essential role in treating AD.

Aβ aggregation can cause excessive oxidative stress in the brain and aggravate neuronal damage, developing into AD-like symptoms (Cheignon et al., 2018). Berberine and jatrorrhizine can achieve neuroprotective effects through antioxidant stress and inhibition of apoptosis (Friedemann et al., 2015; Luo et al., 2017). Ferulic acid (FA) may repair the pathological damage of AD by improving the imbalance of mitochondrial division-fusion kinetics, interfering with oxidative stress and inflammation caused by Aβ aggregation, and then regulating the biological pathways involved in apoptotic programmed cell death (Wang Q. et al., 2019; Wang E. J. et al., 2021). Similarly, chlorogenic acid can control many pathophysiological characteristics of neurodegenerative diseases by improving mitochondrial dysfunction, reducing neuroinflammation and Aβ protein aggregation, and antioxidant stress (Phadke et al., 2021). The improvement of dysfunction, reduction of oxidative stress, and apoptosis may provide a direction for treating AD.

This may be a drug with great potential, as the regulatory effect of plant components of CR on core targets has also been widely reported. We can find detailed data in Table 5. For the IL1B, MAPK8, MMP2, RELA, STAT3, and TGFB1 mentioned in this article. We found that most of the plant components of CR have a down-regulating effect on them, although it is somewhat opposite to the mRNA expression of genes in GEO mentioned in the article, such as MAPK8 decreased significantly in FC and TC. We looked up the literature and found that most of the components were down-regulated to MAPK8, but the overall results were beneficial to our conclusion because the mRNA expression of RELA, STAT3, and TGFB1 increased significantly in AD, and our CR or chemical constituents had a down-regulating effect on them. Therefore, we can speculate that CR can play a role in AD treatment by down-regulating the mRNA expression of IL1B, MMP2, RELA, STAT3, and TGFB1, mediating the HIF-1 signaling pathway, the PI3K-Akt signaling pathway, the Toll-like receptor signaling pathway, and other pathways.

**Table 5:** Analysis of chemical constituents and core targets regulation of CR.

**Components**	**Downregulate**	**References**
Berberine	IL1B, MAPK8,MMP2,TGFB1,RELA,STAT3	Seo et al., 2015; Zhang et al., 2018; Sun et al., 2019
Vanillic acid	RELA,MMP2,STAT3	Kim et al., 2010; Bhavani et al., 2017; Zhu et al., 2019; Park et al., 2020
Palmatine	IL1B, MMP2, TGFB1, STAT3,RELA	Yan et al., 2017; Fan et al., 2020; Ma et al., 2021
Jatrorrhizine	RELA,MAPK8	Jiang et al., 2015
Columbamine	STAT3, MMP2, MAPK8	Bao et al., 2012; Yang et al., 2021
Coptisine	IL1B, MAPK8, RELA,MMP2,STAT3	Wu et al., 2016; Cao et al., 2018; Wang Y. et al., 2021
Ferulic acid	IL1B, MAPK8, RELA,TGFB1,STAT3	Meng G. et al., 2018; Rehman et al., 2019; Yin et al., 2019
Chlorogenic acid	RELA,IL1B,MAPK8,STAT3	Vukelić et al., 2018; Zamani-Garmsiri et al., 2021

## Conclusion

**Figure 11:**
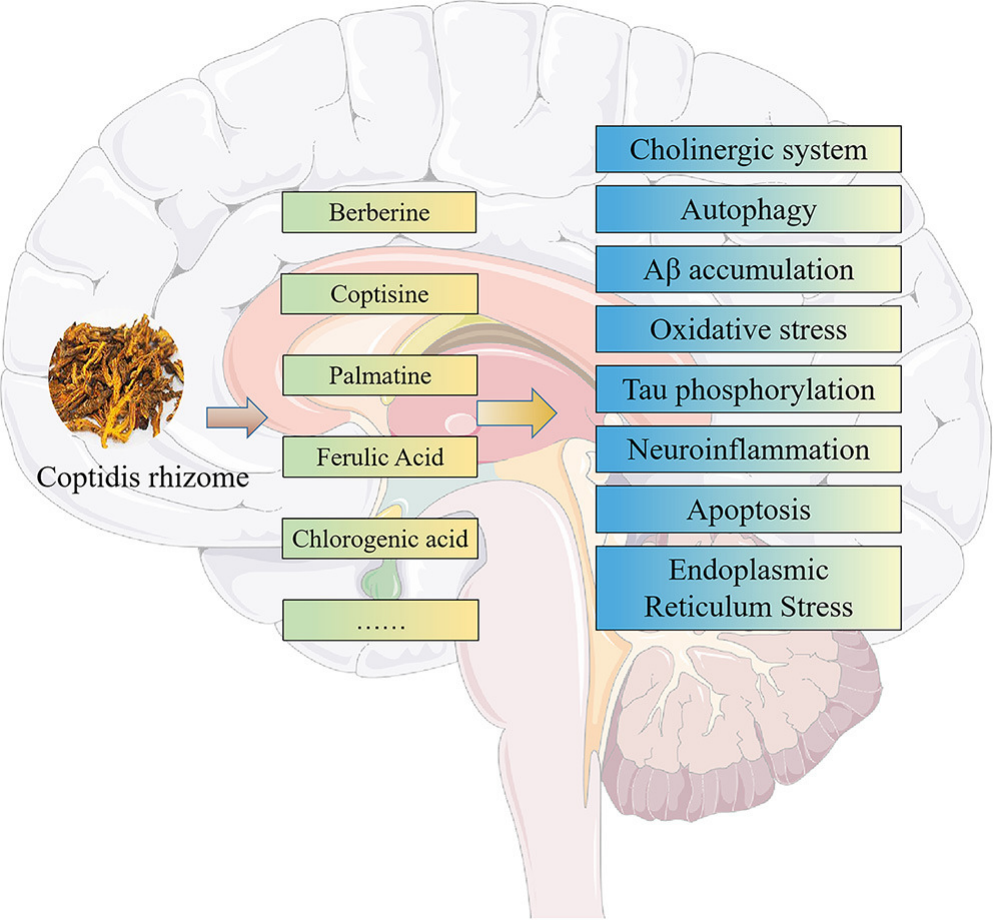
Summary of CR involved in various pathological processes of AD.

To sum up, the components of CR have multi-target pharmacological effects in AD treatment (Table 6; Figure 11), which helps to bring new insight into the best treatment of AD. This paper studied the multi-component, multi-target, and multi-pathway Mechanism of CR in the prevention and treatment of AD by utilizing a network pharmacology strategy. Berberine is expected to become a potentially effective component in the treatment of AD, which may provide a new direction for the current dilemma of AD treatment. Still, the other therapeutic effects of CR active components on AD have yet to be determined.

**Table 6:** CR active ingredients in AD treatment.

**Involved path**	**Ingredient**	**Research model**	**Effective dose**	**Duration**	**Detection index**	**References**
Cholinergic system	Berberine	Intracerebroventricular streptozotocin (ICV-STZ) injected Wistar Rats	50, 100 mg/kg/d	21 days	Acetylcholinesterase activity	De Oliveira et al., 2016
	Berberine	Alcoholic dementia in Wistar Rats	25–100 mg/kg/d	45 days	Cholinesterase activity	Patil et al., 2015
Aβ accumulation	Berberine	N2a/APP695sw cells	0.3, 1, 3 μM	1 day	Aβ, BACE1, AMPK	Zhang et al., 2017
	Berberin	APP/PS1 mice	50 ,100 mg/kg/d	14 days	APP, sAPPα, ADAM10 ,ADAM17, sAPPβ,BACE1, NCT, PS1, Aph-1α, Pen-2	Cai et al., 2018
	Berberine	3 × Tg AD Mice	50, 100 mg/kg/d	4 months	APP, BACE1, Aß1-42	Huang et al., 2017
	Berberine	Human neuroglioma H4 cells	1μM	48 h	APP	Asai et al., 2007
	Coptisine	APP/PS1 transgenic mice; Neuronal pheochromocytoma (PC12) cells	50 mg/kg;10 μM	1 month;5 h	cognition, neuron loss, amyloid plaque formation, Indoleamine 2, 3-dioxygenase(IDO); IFN-γ,IDO	Yu et al., 2015
	Ferulic acid	APP/PS1 mice	30 mg/kg	3 months	Cerebral Aβ deposits,Aβ1-40 and Aβ1-42 levels, APP cleavage,	Mori et al., 2019
Tau phosphorylation	Berberine	Wistar Rats injected with STZ by tail vein	200, 100 mg/kg/d	10 weeks	PI3K/Akt/GSK3β, p-tau (Ser202 and Ser404)	Wang et al., 2018
	Berberine	Calyculin A-induced Cytotoxicity and Tau Hyperphosphorylation in HEK293 Cells	20 μg/ml	1 day	P-tau (Ser198/199/202, Ser396, Ser404, Thr205, Thr231)	Yu et al., 2011
	Ferulic Acid	Injecting Aβ1-42 into the lateral ventricle KM mice	0.1 and 0.4 g/kg ig	5 days	Tau; pS396 protein phosphorylated, total Tau protein and S396	Wang Q. et al., 2019
Neuroinflammation	Berberine	LPS induced learning and memory deficit in the Wistar rats	10, 50 mg/kg/d	7 days	NF-κB, TLR4, TNFα, IL-6, AChE, MAPK	Sadraie et al., 2019
	Berberine	Aβ induced inflammatory response in primary microglial and BV2 cells	1,1.2,5 μM	30 mins	IL6,MCP-1,COX2,iNOS,NF-kB,IKBα,JNK,ERK,AKT	Jia et al., 2012
	Ferulic acid	Injected KA into hippocampus CA1 region KM mice	20, 40, and 80 mg/kg	30 days	IL-1β, IL-6, TNF-α	Rui et al., 2018
Oxidative stress	Berberine	APP/PS1 mice	50, 100 mg/kg/d	14 days	GSH, lipid peroxidation, p-tau	He et al., 2017
	Berberine	Glutamate-induced oxidative stress and apoptosis in PC12 and N2a cells	50~1000 μM	1 day	ROS, GSH, SOD, DNA fragmentation	Sadeghnia et al., 2017
	Jatrorrhizine	The cortical neurons exposure to 50 μM H_2_O_2_ for 12h	50Mm	12 h	Bcl-2, Bax, caspase-3, MMP,ROS	Luo et al., 2017
Autophagy	Berberine	3 × Tg AD Mice	50, 100 mg/kg/d	4 months	LC3-II, beclin-1, hVps34, cathepsin-D, P62, Bcl-2	Huang et al., 2017
	Chlorogenic Acid	Aβ25-35-induced SH-SY5Y neuron injury and cognitive deficits model in APP/PS1 mice	CGA (3.125, 6.25, 12.5, 25, or 50μM); 40 mg/kg	1 or 2 day; 6 months	LC3B-II/LC3B-I, p62/SQSTM, beclin1 and Atg5, cathepsin D, mTOR, p-mTOR P70S6K, p-p70s6k and TFEB	Gao et al., 2020
Endoplasmic Reticulum Stress	Berberine	3 × Tg AD mice	50, 100 mg/kg/d	4 months	APP, BACE1, PERK, TRAF2, JNK, Bcl-2, Caspase-12, eIIF2α	Liang et al., 2021
	Berberine	APP/PS1 transgenic mice	260 mg/kg	3 months	APP,BACE1,GSK3,Tau, PERK,eIIF2α	Wu et al., 2021

## Data Availability Statement

The datasets presented in this study can be found in online repositories. The names of the repository/repositories and accession number(s) can be found in the article/Supplementary Material.

## Ethics Statement

The authors declare that the procedures followed were by the relevant clinical research Ethics Committee's regulations and those of the Code of Ethics of the World Medical Association (Declaration of Helsinki).

## Author Contributions

X-wY integrated the data and wrote the manuscript. S-qC and L-jX executed the literature search. X-fX and H-lW implemented corrections in the manuscript. X-rL conceptualized and designed the experimental plan. All authors contributed to the article and approved the submitted version.

## Funding

This study was supported by the National Key Research and Development Program of China (No. 2019YFC1711500) and the National Natural Science Foundation of China (No. 81973480).

## Conflict of Interest

The authors declare that the research was conducted in the absence of any commercial or financial relationships that could be construed as a potential conflict of interest.

## Publisher's Note

All claims expressed in this article are solely those of the authors and do not necessarily represent those of their affiliated organizations, or those of the publisher, the editors and the reviewers. Any product that may be evaluated in this article, or claim that may be made by its manufacturer, is not guaranteed or endorsed by the publisher.

## Abbreviations

AD: Alzheimer's disease
CR: Coptidis Rhizoma
OB: bioavailability
DL: Drug Like
MCODE: Molecular Complex Detection
MCC: Maximal Clique Centrality
TCM: Traditional Chinese Medicine
TCMSP: Traditional Chinese Medicine Database and Analysis Platform
GO: Gene Ontology
KEGG: Kyoto Encyclopedia of Genes and Genomes Pathway
PPI: Protein-protein Interaction
Aβ: amyloid beta.
