# Supplementary material for: Network Pharmacology-Based Strategy to Investigate the Pharmacologic Mechanisms of Coptidis Rhizoma for the Treatment of Alzheimer's Disease
Source: Front Aging Neurosci. 2022 Jun 21;14:890046. doi: 10.3389/fnagi.2022.890046 (PMC9252849; doi:10.3389/fnagi.2022.890046)
Supplement: Supplementary file 1 [file Data_Sheet_1.docx]

**Identified 6 alkaloids from Coptidis Rhizoma based on HPLC**

Xian-wen Ye^1,2^, Hai-li Wang^1^, Shui-qing Cheng^1^, Liang-jing Xia^2^, Xin-fang Xu^1,2*^, Xiang-ri Li^1,2*^

### **1 Experimental material**

### **1.1 Instrument**

FW-100 high-speed universal pulverizer (purchased from Beijing Kewei Yongxing instrument Co., Ltd.); Waters 2489 high-performance liquid chromatography; AligentZorbaxSB-C18 (4.6mm × 250mm, 5 μ m) column; KQ-600DE CNC ultrasonic cleaner (purchased from Kunshan Ultrasonic instrument Co., Ltd.); Kewei water bath pot (purchased from Beijing Xingde Jingyi Technology Co., Ltd.).

**1.2 Reagent**

Chromatographic pure methanol (Fisher Company, USA), Wa purified water (purchased from Hangzhou Wa Group Co., Ltd.) the other reagents are analytically pure. Reference substance: Berberine hydrochloride (PS020505), Jatrorrhizine hydrochloride (PS010630), Palmatine hydrochloride (PS020011), Columbamine hydrochloride (PS020491), Coptisine hydrochloride (PS000605), Epiberberine hydrochloride (PS020526) were bought in Chengdu Pusi Biotechnology Co., Ltd.

**2 Determination method**

Aligent Zorbax SB-C18 (4.6mm × 250mm, 5 μ m) column, A-30mmol/L ammonium bicarbonate (containing 1mL triethylamine and 7 mL ammonia per 1000 mL ammonium bicarbonate solution) as mobile phase, B-acetonitrile, gradient elution (0~15 min, 10% B; 15 min~25 min, 10%~20% B; 25 min~45 min, 20%~30% B; 45 min~50 min, 30%~35% B, 50 min~65 min, 35%~100% B. 65 min~70 min, 100% B. The flow rate was 1.0ml / min, the detection wavelength was 270nm, the column temperature was 30 ℃, and the injection volume was 10 μ L.

**2.2 Preparation of reference solution**

Precise weighing of jatrorrhizine hydrochloride 5.85 mg was placed in a 10 mL volumetric flask and fixed volume with methanol to the scale to obtain solution A. 6.55 mg Columbamine hydrochloride was placed in a 25mL volumetric flask and fixed volume to the scale with methanol to obtain solution B. Berberine hydrochloride of 31.64 mg, Coptisine hydrochloride of 10.00 mg, Palmatine hydrochloride of 10.71 mg and Epiberberine hydrochloride of 6.63 mg was put into 25 mL volumetric flask, 5mL B and 5 mL C solution were added, and then fixed volume to scale with methanol to obtain a mixed standard solution. The mass concentrations of Berberine hydrochloride, Coptisine hydrochloride and Palmatine hydrochloride, Epiberberine hydrochloride, Jatrorrhizine hydrochloride, Columbamine hydrochloride were 1265.6 μ g / mL, 400 μ g / mL, 428.4 μ g / mL, 252 μ g / mL, 117 μ g / mL and 52.4 μ g / mL respectively.

**2.3 Preparation of test solution**

Take about 0.1 g of the sample, weigh it accurately, put it in a conical bottle with stopper, accurately add methanol-hydrochloric acid solution 50 mL, weigh it and then treat it with ultrasonic for 30 minutes. After weighing again, make up for weightlessness and shake well, centrifuge, and get.
